# Ecological restoration should be redefined for the twenty‐first century

**DOI:** 10.1111/rec.12554

**Published:** 2017-06-29

**Authors:** David M. Martin

**Affiliations:** ^1^ Atlantic Ecology Division, Office of Research and Development U.S. Environmental Protection Agency 27 Tarzwell Drive Narragansett RI 02882 U.S.A.

**Keywords:** decision‐making, definitions, restoration goals, social values

## Abstract

Forty years ago, ecological restoration was conceptualized through a natural science lens. Today, ecological restoration has evolved into a social and scientific concept. The duality of ecological restoration is acknowledged in guidance documents on the subject but is not apparent in its definition. Current definitions reflect our views about what ecological restoration does but not why we do it. This viewpoint does not give appropriate credit to contributions from social sciences, nor does it provide compelling goals for people with different motivating rationales to engage in or support restoration. In this study, I give a concise history of the conceptualization and definition of ecological restoration, and I propose an alternative definition and corresponding viewpoint on restoration goal‐setting to meet twenty‐first century scientific and public inquiry.

**Conceptual Implications**

- Ecological restoration is based on using ecological knowledge to understand how ecosystems work and to use that understanding to recover ecosystem conditions that maintain their structure and function.
- Ecological restoration is a mainstream concept with dual social and scientific roles that reflect modern scientific and public views about the many ways that nature is valuable and sustains us. Yet we currently lack a definition that emphasizes the duality of ecological restoration.
- I propose an alternative definition that balances modern views about what ecological restoration does—it aims to recover ecosystem conditions—and why we restore ecosystems—to reflect common values and beliefs—to inspire restoration in the twenty‐first century.

## Introduction

*Restoration Ecology* will be commemorating 25 years next year. In 2003, this journal expanded its editorial scope to receive any contributions to understanding restoration, regardless of scientific discipline (Allen 2003). Many publications recognize the social dimensions of ecological restoration; they include articles (Higgs 1997; Lackey 1998; Hobbs et al. 2004), books and other edited volumes (Gobster & Hull 2000; Higgs 2003; Aronson et al. 2007; Egan et al. 2011; Clewell & Aronson 2013), and guidance documents, including those from the Society for Ecological Restoration (SER; Gann & Lamb 2006; 2016a, 2016b), Parks Canada and the Canadian Parks Council (Parks Canada 2008), The Nature Conservancy (TNC 2016), the United Nations Convention on Biological Diversity (CBD 2016), and the International Union for Conservation of Nature (Keenleyside et al. 2012). These contributions explicitly acknowledge that setting goals for restoration requires people to articulate what it is about ecosystems that are valued. In light of these and related changing viewpoints on the concept, science, and practice of ecological restoration (Suding et al. 2015; Hobbs 2016; Richardson & Lefroy 2016; Rohwer & Marris 2016), how we define ecological restoration needs revisiting.

Definitions that pertain to mainstream concepts should reflect how we view those concepts. Currently, the most accepted definition of ecological restoration was published in the SER Primer on Ecological Restoration (hereafter referred to as Primer; SER 2002):

Ecological restoration is the process of assisting the recovery of an ecosystem that has been degraded, damaged, or destroyed.

This definition promotes the concept of ecological restoration in a specific way. Yet the phrase “ecological restoration” is mainstream (Clewell & Aronson 2013) and therefore, in concept, ecological restoration reaches farther than ecosystem recovery. This is in contrast to, for example, the phrase “restoration ecology,” which has carried a specific conceptualization through time as a field of science associated with the practice of restoring ecosystems.

How we view ecological restoration is 2‐fold: (1) we view it by what restoration does—it assists in the recovery of ecosystem conditions that maintains ecological structure, process, and function and (2) we view it by why we restore ecosystems—to achieve common values and beliefs. This rationale is paraphrased from a popular marketing mantra: “people don't buy what you do; they buy why you do it” (Sinek 2010). The SER International Standards document (McDonald et al. 2016*a*) emphasizes that the phrase “ecological restoration” is commonly used to describe these two views, but why is the concept defined to reflect what it does and not why we do it?

The purpose of this study is to combine these two views and redefine ecological restoration to meet modern scientific and public inquiry. This approach aims to balance our rationalization of the promise, or what restoration does, and appeal, or why we restore ecosystems, of ecological restoration. I start by unraveling the historical conceptualizations and alternative definitions of ecological restoration. I document the emergence of the social dimensions of ecological restoration, and I attempt to capture mainstream social and scientific aspects with a proposed alternative definition and corresponding viewpoint on how it could guide restoration goal‐setting. This frame of reference will allow scientists and other groups, including governmental and nongovernmental organizations, indigenous groups, restoration practitioners, and other community associations to expand their views on ecological restoration and to incorporate those views into environmental management and decision‐making.

## Historical Context for Ecological Restoration (1970s–1990)

Ecological restoration has a deep history. Although there are centuries of documented stories and examples of humanity's nurturing interaction with the natural environment, attention to the practice of land and water restoration grew in the 1970s (Hall 2005; Jordan & Lubick 2011). The concept of ecological restoration was presented in definition through a descriptive, natural science lens. In their book, *The Restoration of Land*, Bradshaw and Chadwick (1980) offer an early definition:

In this book restoration is used as a blanket term to describe all those activities which seek to upgrade damaged land or to re‐create land that has been destroyed and to bring it back into beneficial use, in a form in which the biological potential is restored.

In *Restoring the Earth*, Berger (1987) offers a related definition:

Natural resource restoration is a process in which a damaged resource or region is renewed. Biologically. Structurally. Functionally.

Early definitions promoted a science‐based promise to use ecological knowledge to fix degraded ecosystems.

Diamond (1985) was among the first to draw attention to the social dimensions of restoration as he reflected on discussions at the University of Wisconsin‐Madison Arboretum symposium in 1984:

First, no community on Earth has escaped the direct or indirect effects of man, so which is the ‘natural community’ that one would seek to restore?

Diamond's inquiry was followed by more symposia in the United States (University of California, Berkeley, 1988; first annual SER meeting in Oakland, CA, 1989) and abroad (Venezuela, Hungary, India, Jordan 1988*a*) and other writings on the subject (e.g. Diamond 1987; Morrison 1987; Jordan 1988*b*; Sayen 1989), which called for better articulations of what the practice of restoration was about, who it was about, and how success could be measured. Although it may have been used earlier, the phrase “ecological restoration” appeared in print in a 1984 article in *Restoration and Management Notes* (Jordan & Lubick 2011). It was not commonly used until the SER formed in 1988.

## The Social Context of Ecological Restoration (1990–2000s)

Extraordinary progress in the conceptualization of ecological restoration occurred between 1990 and 2000. At the fourth annual conference of the SER in 1992, Alex Wilson led a series of contentious discussions on scientific and social issues surrounding restoration (Higgs 2003). In a footnote to an early commentary on the subject of social dimensions, Higgs (1994) wrote:

Ecological restoration is the total set of ideas and practices (social, scientific, economic, political) involved in the restoration of ecosystems.

Higgs emphasized ecological restoration as a process where the means, or restoration goals, cannot be detached from the ends, or conditions of the resulting ecosystem. This was followed by Cairns (1995), Light and Higgs (1996), Higgs (1997), more symposia on the subject (Egan et al. 2011), and the edited volume by Gobster and Hull (2000), all of which recognized an embedded social context in ecological restoration. The commentary in Hobbs et al. (2004) firmly established that common values were implicitly used to set restoration goals. As many have described, those values can be inherent or instrumental and can cover social, environmental, economic, cultural, moral, political, or religious contexts and meanings.

Although these and other contributions did not summarize this notion into a standardized definition of ecological restoration, it is not that people have not tried. In fact, the SER Board considered many alternative definitions in the early 1990s before asking Dennis Martinez to co‐chair the SER Science and Policy Working Group, along with Eric Higgs, to deliberate repeatedly over definition; it took 6 years, between 1995 and 2001, and many alternative definitions before the group agreed on a definition that settled some contentious phrases of the past (i.e. “ecological integrity,” “indigenous ecosystem”) and achieved wide acceptance (Higgs 2003). In 2002, the SER Science and Policy Working Group, then chaired by Keith Winterhalder, established an official definition in the first edition to the Primer (SER 2002); it was reprinted in the second edition in 2004 and resonates today.

It is important to note that Davis and Slobodkin (2004) presented a definition that emphasized value:

Ecological Restoration is the process of restoring one or more valued processes or attributes of a landscape.

The United Nations Convention on Biological Diversity recently published a similar definition (CBD 2016):

Ecological restoration refers to the process of managing or assisting the recovery of an ecosystem that has been degraded, damaged or destroyed as a means of sustaining ecosystem resilience and conserving biodiversity.

These definitions place value on ecosystem conditions regarding the maintenance of ecological structure and function more so than on how recovered ecosystem conditions reflect benefits to people (see Connection to Restoration Goal‐Setting).

The aforementioned definitions provide promise but not appeal. They do not explicitly recognize the breadth of modern scientific research on the subject, nor do they admit that social engagement and value‐laden goal‐setting are fundamental parts of the restoration process. On the contrary, contributions from behavioral and environmental economics, ecological economics, education, sociology, psychology, and the decision sciences have led to compelling recognitions of (1) inherent and critical links between ecological restoration and ecosystem services (Holl & Howarth 2000; Loomis et al. 2000; Palmer et al. 2004; Aronson et al. 2007; Chazdon 2008; Benayas et al. 2009; Palmer & Filoso 2009; Aronson et al. 2010; Bullock et al. 2011; Clewell & Aronson 2013; Alexander et al. 2016; and many others) and (2) improvements in public perception, stewardship, morality, and environmental literacy that underlie and accompany restoration (Berger 1987; Miles et al. 1998; Bowler et al. 1999; Gobster & Hull 2000; Graham 2007; Junker & Buchecker 2008; Buijs 2009; Egan et al. 2011; Goleman et al. 2012; Shume 2016; and many others).

## An Alternative Definition of Ecological Restoration

Published discussions over definition diminished gradually after the Primer was issued. Nevertheless, credit should be given to the long unpublished history on the subject that includes decades of conference proceedings, SER committee discussions, and countless discussions among restoration theorists and practitioners (Higgs 2003; Hall 2005; Jordan & Lubick 2011). Some environmental groups have adopted the Primer definition, such as Parks Canada and the Canadian Parks Council, the International Union for Conservation of Nature, and Conservation International (Parks Canada 2008; Keenleyside et al. 2012; Kipp Lanham 2017, Conservation International, personal communication), whereas others have separate but similar definitions that focus on ecosystem conditions, such as The Nature Conservancy (www.tnc.org) and the United Nations Convention on Biological Diversity (CBD 2016), but all provide supporting goal and/or visionary statements regarding common values and beliefs.

In sum, we should be able to explain more precisely how ecological restoration is viewed in its definition, other than by insisting that restoration aims to achieve some characteristics of the resulting ecosystem. One way to do this is by amending the Primer definition:

Ecological restoration is the process of assisting the recovery of a degraded, damaged, or destroyed ecosystem to reflect values regarded as inherent in the ecosystem and to provide goods and services that people value.

This alternative definition remains faithful to the same guidelines and core concepts presented in the Primer and other guidance documents (Gann & Lamb 2006; Parks Canada 2008; Keenleyside et al. 2012; McDonald et al. 2016*a*). Likewise, by recognizing the potential for ecological restoration to foster inherent values (i.e. values that personally or altruistically resonate with people) or utilitarian values (i.e. conventional ecosystem services), it is flexible to cover all of the motivating rationales for people to embrace restoration, as they are defined in Clewell and Aronson (2006). This includes the rationales of global conservation organizations, whose vision and mission statements have evolved to focus on social dimensions (e.g. Conservation International, International Union for Conservation of Nature, The Nature Conservancy, World Wildlife Fund), private corporations, federal and state agencies, indigenous groups, and the many thousands of volunteers and paid practitioners who participate in restoration worldwide.

## Connection to Restoration Goal‐Setting

In addition to balancing emphasis on the resulting ecosystem (structure, process, function) and what we get from that ecosystem (value), the alternative definition could make restoration goal‐setting more robust. Targets and goals for restoration are often indicator‐ or attribute‐based, that is, they refer to what structural or functional ecosystem conditions can be recovered, such as species composition, community structure, ecological complexity, historic continuity, self‐sustainability, and ecosystem resilience (Clewell & Aronson 2013). Shackelford et al. (2013) pointed out that attribute‐based goals do not explicitly incorporate social values. Likewise, Hallett et al. (2013) examined over 200 restoration projects in the Global Restoration Network database and revealed that social values are well‐used, albeit not as much as ecosystem attributes, to develop project goals. Some guidance documents approach goal‐setting using social‐based values and beliefs (e.g. improve biodiversity conservation, improve human livelihoods, empower local people) (Gann & Lamb 2006; TNC 2016), some approach goal‐setting using ecosystem conditions and social values (Keenleyside et al. 2012), whereas others operate on a slightly modified planning hierarchy that foremost acknowledges social values and then identifies ecosystem‐based goals and attributes around those values (Parks Canada 2008).

Why is there such a dichotomy of goal‐setting approaches? One reason is because ecosystem attributes are not goals that motivate the broader achievement of restoration; they do not have direct value. Rather, ecosystem attributes are means toward achieving value‐laden objectives. Nobody is following the same process in part because of a flawed definition of ecological restoration that views ecosystem attributes as valued outcomes.

**Figure 1:**
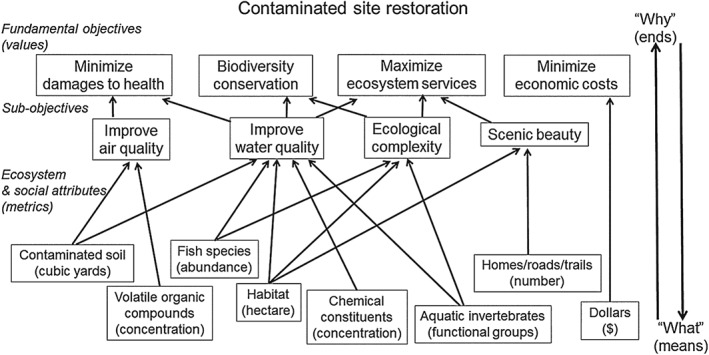
An example objectives hierarchy for restoration goal‐setting.

Decision‐makers, scientists, and other restoration professionals and practitioners should follow a structured, hierarchical goal‐setting process (e.g. Fig. 1; sometimes referred to in the literature as an objectives hierarchy) that is guided by a simple question: “why?” Why is recovering the structure and functionality of an ecosystem important? Because biodiversity, species persistence, and esthetic “naturalness” appreciation are valuable to people. Why is restoring topsoil or vegetated habitat important? Because less sediment is allowed to deposit into rivers. But why is sediment reduction in rivers important? Because people and aquatic organisms desire clear water, which is a characteristic of water quality. Then why is improved water quality important? Because sustainable fish populations are culturally valuable to indigenous people, or perhaps because ecosystem services like recreation, nature viewing, commercial fishing, and safe drinking water are valuable to people.

If we keep asking “why is it important,” we will reach “just because” endpoints in our goal‐setting (Keeney 1992). It is at these endpoints where we realize that restoration aids in the establishment of multiple potential ecosystem or social conditions that can be traced to value‐laden outcomes. In this context, ecosystem attributes are just performance metrics that measure the consequences of value‐laden objectives as they apply to decisions to implement restoration strategies.

This viewpoint is grounded in value‐focused thinking, which is used in structured decision‐making approaches to environmental management (Gregory et al. 2012), and creates a distinct difference between value‐laden restoration objectives, which articulate our concerns and wishes for ecological restoration, and ecosystem attributes, which quantify the level of achievement of the objectives in a representative and measurable manner (Fig. 1). Using this approach, restoration professionals and practitioners are encouraged to decide what they cared about first (“why” in Fig. 1) rather than trying to get what they care about (“what” in Fig. 1) without explicitly stating what it is that they care about (Keeney 1992). Breaking the goal‐setting process down into parts has advantages: (1) it allows the process to be more transparent and documentable, which could control for unintended costs or restoration failures, (2) it allows for roles to be clearly defined, which could control for scientists inserting normative preferences into the process (i.e. Lackey 2016), and (3) it allows for multiple potential goals and objectives, including associated ecosystem and social attributes, to be optimized using decision analytic procedures for integrated decision support (for examples of case studies using the approach, see Gregory & Long 2009; Failing et al. 2013; Robinson et al. 2016).

In conclusion, the explicit union of social and scientific views in the definition of ecological restoration is why the Primer and related guidance documents need revisiting. In doing so, we could allow ourselves to directly connect means with ends, or appeal with promise, and we may discover a more robust goal‐setting structure for ecological restoration. Notwithstanding these opinions, what people value will remain the decision context for ecological restoration. If ecological restoration for the twenty‐first century, or “Restoration v2.0” (Higgs et al. 2014), emphasizes pragmatic goals for human well‐being as a driving force, then explicitly capturing the social and scientific contexts of ecological restoration in its definition may broaden the framing and practice of restoration worldwide.
